# A motion sensing-based framework for robotic manipulation

**DOI:** 10.1186/s40638-016-0056-9

**Published:** 2016-12-09

**Authors:** Hao Deng, Zeyang Xia, Shaokui Weng, Yangzhou Gan, Peng Fang, Jing Xiong

**Affiliations:** 1 Shenzhen Institutes of Advanced Technology, Chinese Academy of Sciences, Shenzhen, China; 2 Laboratory of Human-Machine Intelligence-Synergy Systems, Chinese Academy of Sciences, Shenzhen, China

**Keywords:** Human–machine interaction, Motion sensing, Gesture recognition, Robotic manipulation

## Abstract

To data, outside of the controlled environments, robots normally perform manipulation tasks operating with human. This pattern requires the robot operators with high technical skills training for varied teach-pendant operating system. Motion sensing technology, which enables human–machine interaction in a novel and natural interface using gestures, has crucially inspired us to adopt this user-friendly and straightforward operation mode on robotic manipulation. Thus, in this paper, we presented a motion sensing-based framework for robotic manipulation, which recognizes gesture commands captured from motion sensing input device and drives the action of robots. For compatibility, a general hardware interface layer was also developed in the framework. Simulation and physical experiments have been conducted for preliminary validation. The results have shown that the proposed framework is an effective approach for general robotic manipulation with motion sensing control.

## Background

Robots have found an increasingly wide utilization in all fields, and they move objects with blurring speed, repeat performances with unerring precision and manipulate tasks with high dexterity. Robots have long been imaged as mechanical workers, cooperating with or even replacing people [1]. Yet the situation is, to data, robots have been very successful at manipulation in controlled environments such as in a factory or laboratory. When outside of the controlled environments, they normally perform tasks when operating with human [2]. Although the latest robotic researches would handle the situations with a sophisticated sensing and intelligent system [3], most robots are still under a typical “teach-pendant operating” pattern to get knowledge about current environment configurations.

**Fig. 1:**
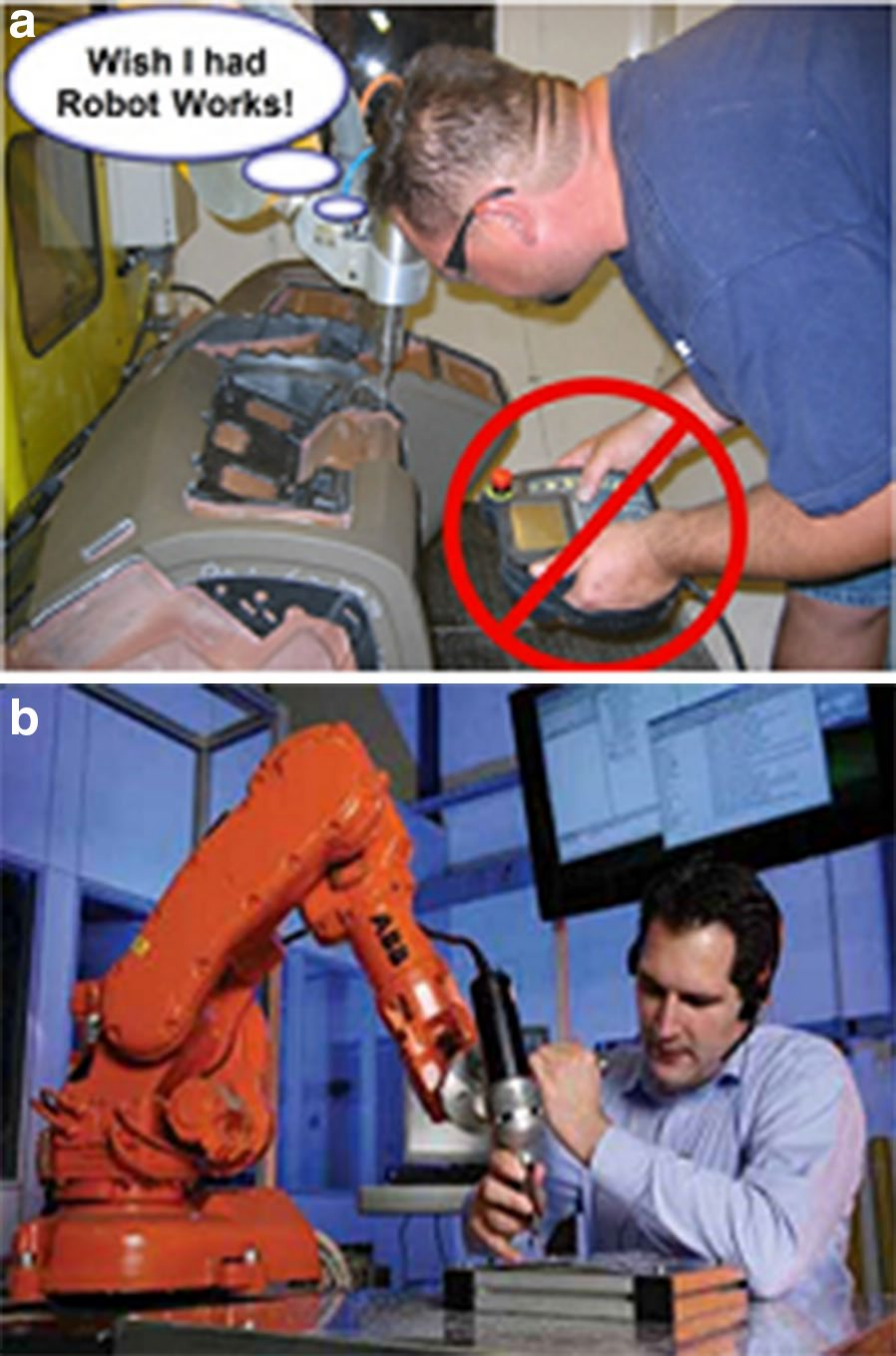
Typical operation patterns. **a** Teach-pendant operation with hand–eye coordination and fully focus [5] and **b** “lead-by-the-nose” operation for paint spraying [6]

Typical teach-pendant is a handheld control unit equipped with buttons or joysticks to manually send the robot to desired positions, a screen to display robot states, and a large red emergency stop button. In teach-pendant process, operators need to stand in certain nearby area, hold the control unit and move robot manually; meanwhile, special focus should be paid on to avoid collision and identify the arrival at targeted position, as shown in Fig. 1a. The whole procedures require operators with high technical skills training, and high hand–eye coordination; otherwise, it would be so extremely easy to lead serious collision damage to operators or equipments. Differing from the active teach-pendant operation mode, “lead-by-the-nose” is a passive technique, in which, controller will de-energize the robot joints and allow users to drag and move the robot by hand to any desired positions or even record paths [4], as shown in Fig. 1b.

However, both techniques mentioned above are hardware dependent and controller specific, which definitely increase learning and training costs when adapting to new types of robotic system. In final analysis, all these issues come down to a matter of lacking user-friendly but general human–machine interaction interface. Recent researches have shown that the motion sensing technology enables human–machine interaction in a novel and natural interface using gestures or spoken commands. And latest revolutionary motion sensing devices, like Kinect [7] and Leap Motion [8], are boosting wider applications of motion sensing technology from gaming to robotics [9, 10].

Some approaches have put forward that the motion sensing data can control the action of a robot, where robot will initiate human movements, or learn actions from human [11], and this motion sensing is usually at the crossroad between gesture recognition and skeleton tracking. In practice, sometimes, we only interested in the pose of hand, which can be used to directly drive the end effector of a robot, or in some other cases, we may also need to command robot in a jointed mode which requires joint angles date captured from skeleton tracking. In previous studies, numbers of works have been proposed on use of motion sensing input devices for robotic applications [12].

However, most of them were focusing on specific cases, which usually integrated a certain type of sensors, Kinect, Leap Motion, or et al., for a targeted robot system configuration. And there is no systemic solutions published for general motion sensing-based robotic manipulation. So our objects in this research are: (1) providing a modular and easy plugin-and-play framework to connect between any available motion sensing input devices and robotic systems. (2) Simplifying the hardware integration by proposing a driver update pattern.

The rest of this paper is organized as follows. “Framework architecture design” section describes the architecture design of our proposed framework. “Motion sensing commands” section shows how to create the motion sensing commands for robotic manipulation. “Framework core” section depicts the control core for robotic manipulation. “General hardware interface” section depicts realization of hardware interface for hardware abstraction. “Implementation and experiments” section demonstrates implementation and experiments of the proposed framework. “Conclusion” section summarizes our study. And “Discussion and future work” section starts discussions about this research and works out the future work.

## Framework architecture design

For motion sensing manipulation, the proposed framework should be the characteristic of: (1) skeleton tracking or gesture capture, especially the poses and joint angles in the chosen sensing area. (2) Mapping from human movements to robot actions. (3) Compatibility with varied hardware, including motion sensing devices and robots.

To meet these requirements, our design philosophy of the framework is to develop as distributed and modular as possible, so that it could be hardware independent and easily configured. The foundation of our framework is laid on the powerful robot operation system (ROS) [13], which adds value to the development of robotics projects and applications with open-sourced packages, libraries and tools.

**Fig. 2:**
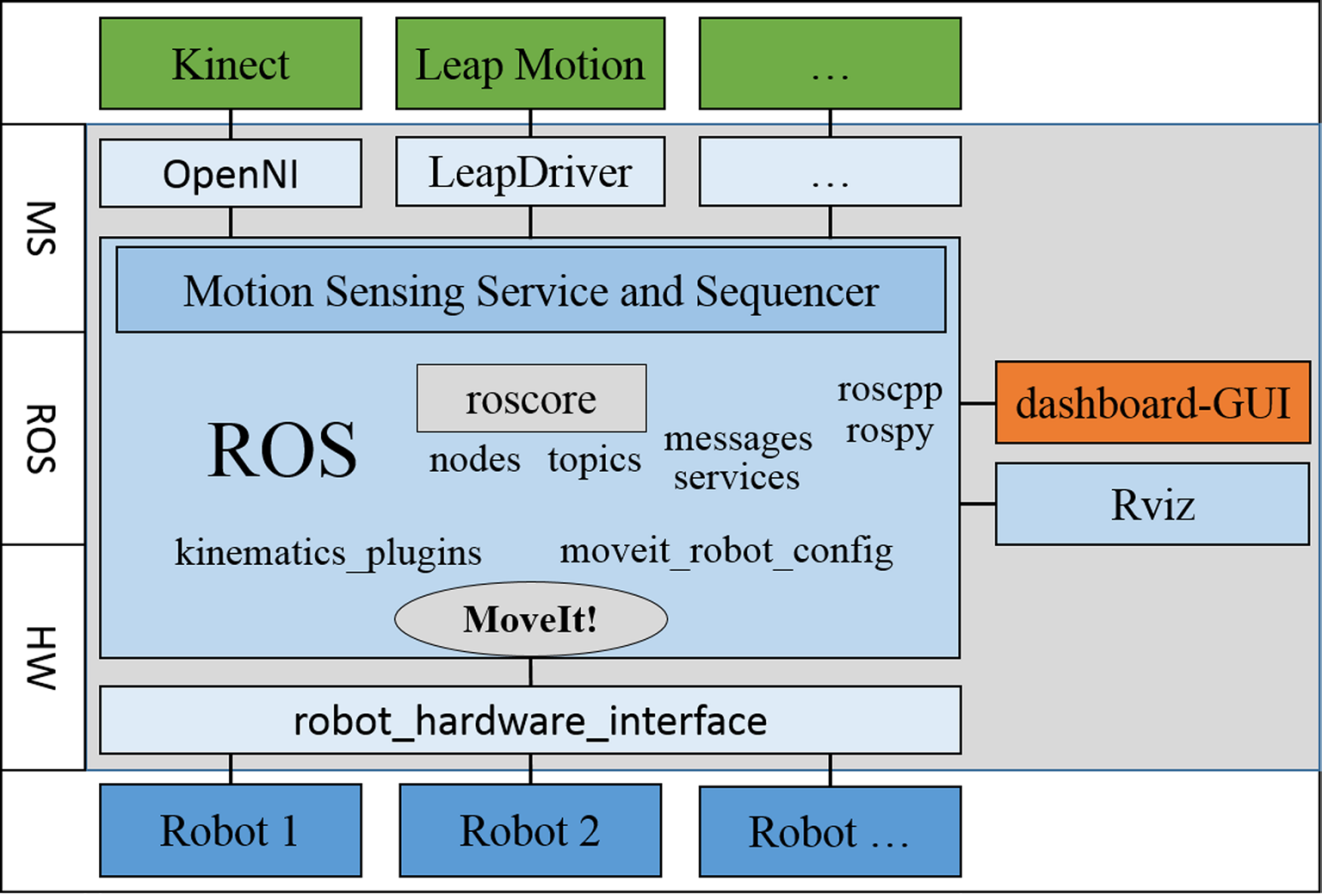
Framework architecture designed. Three main functional layers, the MS-motion sensing layer, ROS layer and the HW-hardware layer

Therefore, the framework is designed in a three-layer structure, which comprises the motion sensing layer, ROS foundation and hardware interface layer, as given in Fig. 2. This three-layer structured framework is targeted to provide an effective approach for general robotic manipulation.

The first motion sensing (MS) layer provides drivers for sensors and manages the raw commands captured from skeleton tracking and gestures. Note that the development of this layer is based on ROS communities and official SDK, while our main contribution is to design the integration of drivers and the distributed and modular designed motion sensing service and sequencer, which allows users to request poses or joint angles data from motion sensing input devices more friendly. Existing services can be modified, or new services can be added very easily in ROS layer to adapt for the requirement of customized manipulation task.

The ROS foundation layer is at the heart of the proposed framework, which can be called the framework core. Functionally, this layer can be divided into four modules, which are the information management module, model management module, manipulation control module and visualization module. Developed on ROS platform, this layer creates the middleware between motion sensing devices and the real robots.

The hardware interface (HW) layer is a hardware communication interface that talks and listens to the controller of physical robots. With communication protocols, it can build a service-request mode between the framework and the actual robots. The main advantage of this layer is, based on ROS industrial, the integration can be established on any hardware with communication protocol supported.

## Motion sensing commands

In this section, we will focus on how to understand the intent of the operator and create motion sensing commands for robotic manipulating. Even though robots can be operated in end effector mode and joint mode, for motion sensing commanding, end effector mode will be more compatible regardless of different DOFs configuration. In our current study, we only focus on tracking of hand articulations.

### 3D tracking of hand articulations

**Fig. 3:**
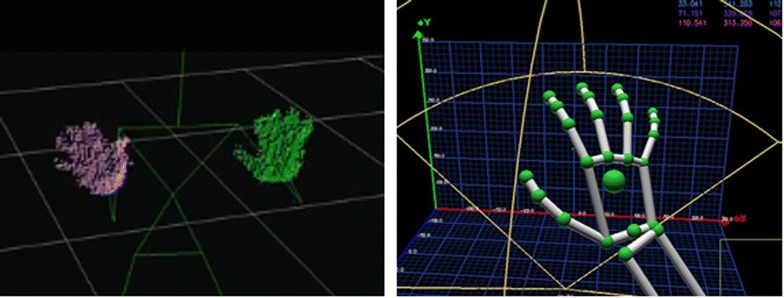
3D tracking of hands with position, orientation and full articulation. **a** Two hands tracking from Kinect, displayed in Rviz and **b** hand skeleton tracking from Leap Motion, displayed in LeapCommandCenter

Tracking articulated objects is an interesting and hot research problem, and latest revolutionary motion sensing devices are boosting wider applications of this technology from gaming to robotics. Among them, 3D tracking of human hands is the most efficient and straightforward. We can acquire the 3D position, orientation and full articulation of a human hand. And in ROS communities, MIT Kinect demos [14] provided purely hand and finger detection, and Leap Motion SDK [15] also supported for hands skeleton tracking, as shown in Fig. 3.

**Fig. 4:**
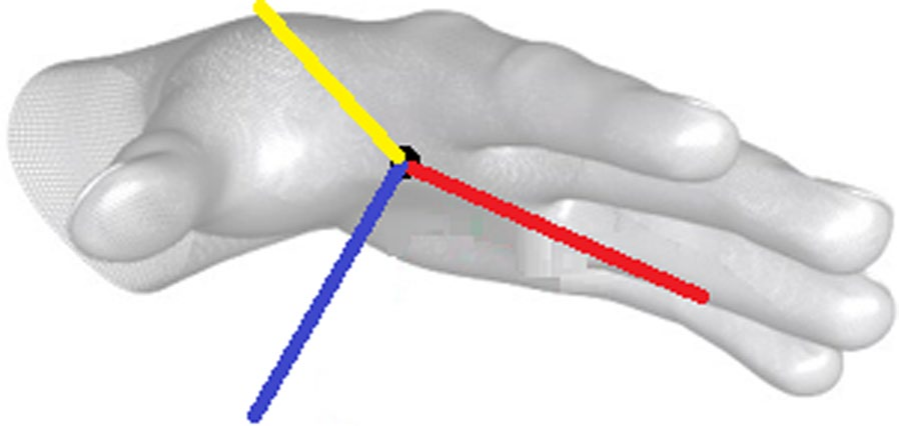
Tracking model definition. Hand palm tracking in our current motion sensing framework

Here we defined a universal hand model for tracking as given in Fig. 4. All motion sensing devices will be capable of tracking the hand palm and provide the following information: hands numbers, hand types, position of each hand palm, and direction of each hand palm. All the information is standardized in a ROS message type *motion_sensor/hand.msg* as defined in Table 1 and can be published from motion sensing service in a given topic */motion_sensor/hand*.

Based on the device drivers from ROS communities, we modified the original interfaces and remapped the data pipelines into the defined message type. Note that in our framework not all official functions of the devices are supported, but it is enough for motion sensing robotic manipulating, and also this capability can be easily extended. In this layer, a motion sensing and sequencer module was developed, which will bind multiple motion sensor input, standardize and integrate with ROS layer. As whole framework is designed modularly, when adopted new sensors, this layer can be packaged and installed as driver and later easily updated for new features over the air using the built-in rosinstall mechanism.

**Table 1:** Standardized message definite for hand information

motion_sensor/hand.msg	
std_msgs/Header	header
uint32	hands_num
string[]	hand_typer
geometry_msgs/Point	position
geometry_msgs/Quaternion	orientation

### Motion sensor data processing

Even though numerous palm pose can be captured and acquired from motion sensing devices, there are still some theoretically interesting problems, space registration, sensor data filtering, and dynamic response.

**Fig. 5:**
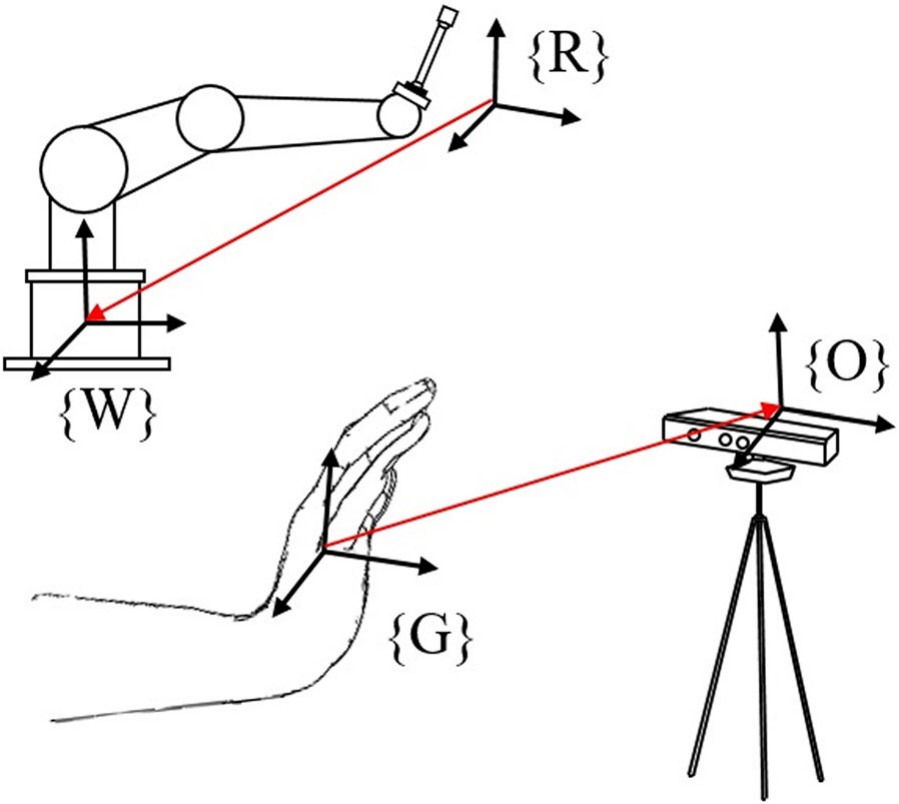
Space registration. The coordinate systems in motion sensing-based manipulating operation

To satisfy quick trajectory tracking, the hand space {*G*}, motion sensing device space {*O*} and the robot space {*R*} need to be mapped to realize registration. Since we have no additional optical measuring device to build this transformation relationship during operation, we make full use of dynamic time warping (DTW) to calculate every pose distance between two hand gestures to measure the minimal motion similarity [16] between end effector of the robot and the hand. Thus, we avoid building the transformation relationship between coordinate system, but try to map the dynamic distance matrix of hand palm in motion sensing device space {*O*} to distance matrix of end effector in world space {*W*}, as shown in Fig. 5. Source motion created by hands will be speed-reduced in a given ratio and imitated by the robot.

**Fig. 6:**
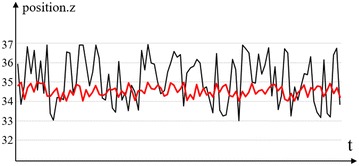
Sensor data filtering. Palm position in *z* direction location plotted for a certain time when holding hand in one fixed pose where the *black line* is the raw sensor data and *red line* is the sensor data after filtering

Motion sensing devices are so acute that manipulation operation may easily fail due to sensor noise or natural hand shaking. In Fig. 6, the plotted hand palm position in *z* direction from raw data shows a slight fluctuation, which will definitely lead robot to tremble in the corresponding axial direction. Additionally, uncontrolled hand movement, especially when hand moves into and departs from the sensing area, may also cause abnormal action of the robot. When choosing the filter, we value its response performance. So the simplest way we used is a mean filtering. The red line in Fig. 6 is the result of sensor data after filtering, in which the queue size is set as 10. And for uncontrolled hand movement, we set an optimal threshold to ignore huge pose changes in a short time.

**Fig. 7:**
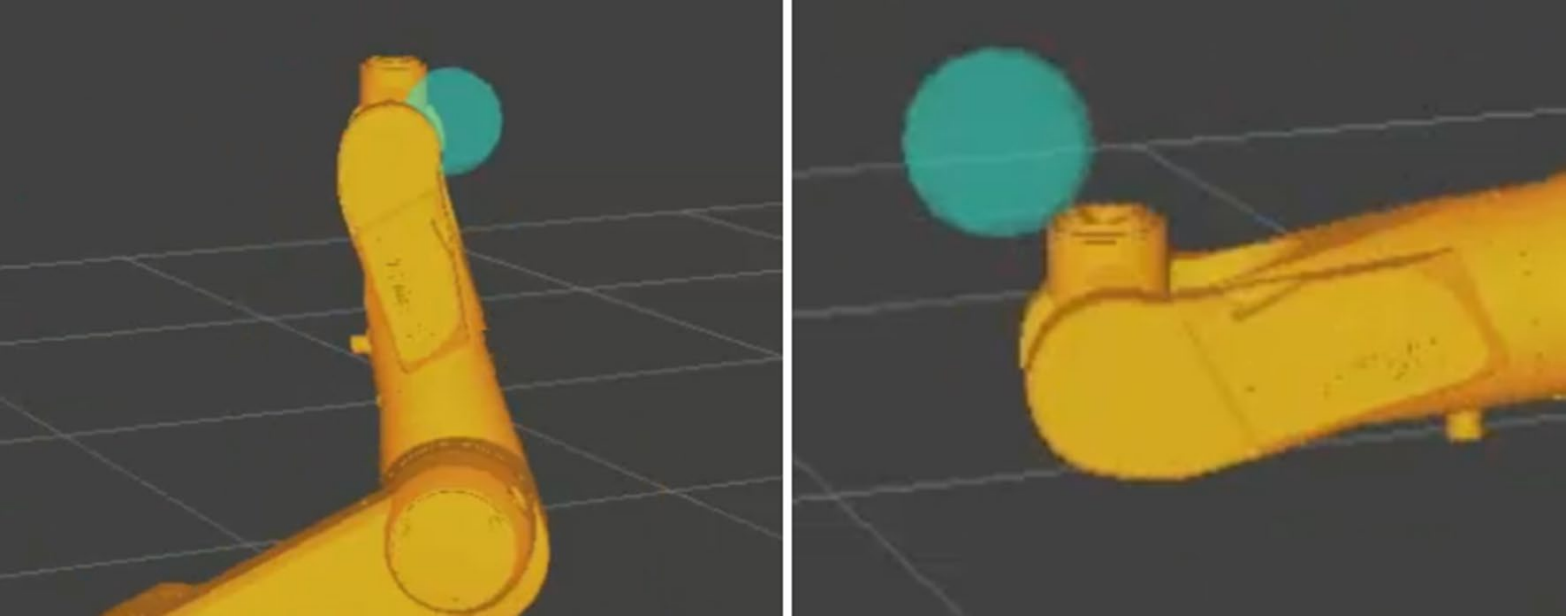
Motion sensing command delay. Display the raw palm position with ball marker and simulate the corresponding arm action

However, in preliminary experiments, we found out that robot action would wait for a noticeable delay when the sensor is triggered, as shown in Fig. 7. Theoretically, this delay mainly comes from the sensor SDK algorithm, data filtering and mapping from gestures to robot commands. For Kinect, the algorithm delay is recorded and estimated to be five frames, and also the first and last 10 frames of the raw data were ignored by setting the optimal threshold for huge pose changes in a short time. For data filtering delay, it is difficult to strike the right balance between the stability and dynamic response.

In our framework, max rate of raw sensor data acquisition is in 30 frames/s. The real-time computation of data filtering and coordinate mapping is costly. Motion sensors will never be real-time responsive, and it is very important that the code is running the data read from the sensor and the position command asynchronously. In our study, we took stamp.nsecs in the published sensor message to compare with robot states and drop outdated frames until the action is done.

### Motion sensing commands publish

After getting intents of operator, the layer need tell the information to robot controller. Since motion sensing commands are specifically 6D-pose of the hand palm, the information is managed in *geometry_msgs/Pose* message type and published to global /*tf*.

**Fig. 8:**
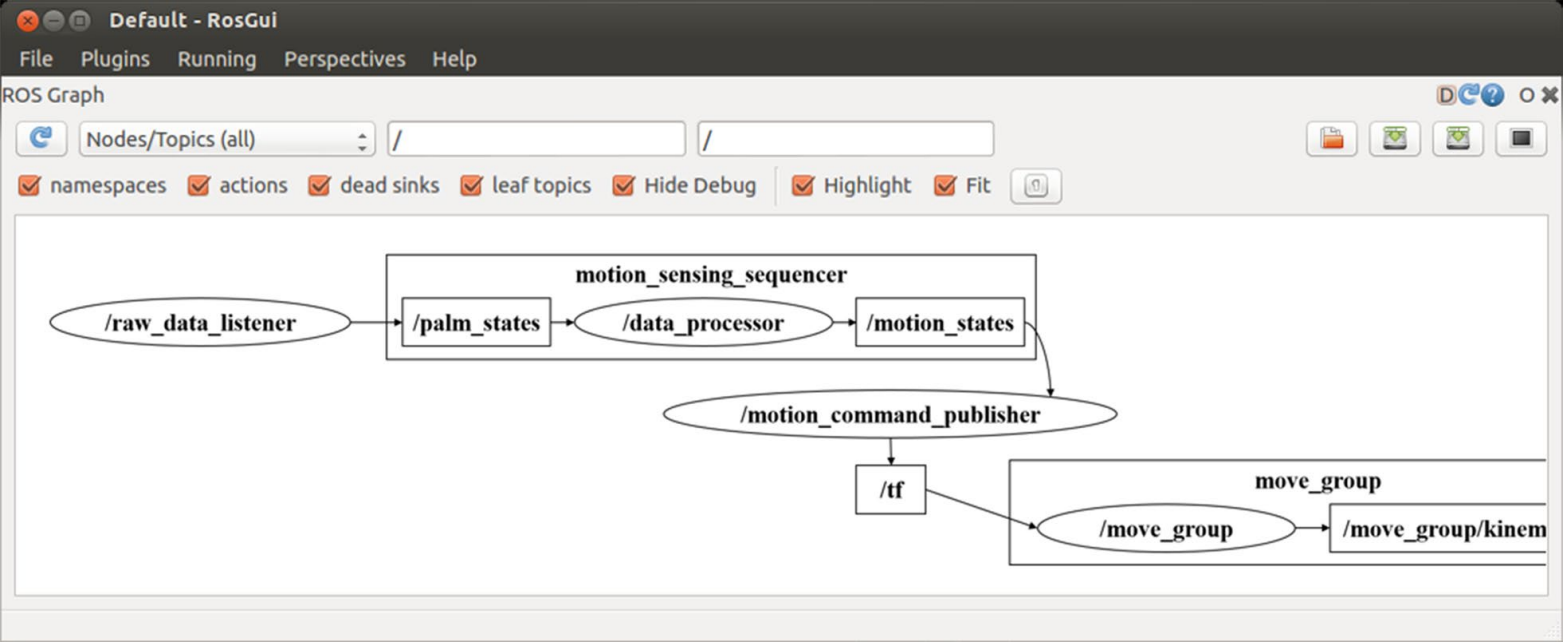
Nodes and topics in motion sensing layer. The sequencer subscribes raw sensor data published by sensor driver, then performs data processing and publishes captured motion states

The relationship of nodes and topics in MS layer can be created using rqt_graph tool as shown in Fig. 8.

## Framework core

As the framework core, ROS foundation layer consists of four modules and plays as the middleware combining all software and hardware together. On functional level, those modules are responsible for information management, model management, manipulating control and visualization.

### Information management

In a typical ROS system, the roscore process acts as the central manager of all nodes and establishes point-to-point communication between nodes in topics or services paradigms. A ROS topic can publish and subscribe messages to certain topics, resulting in building a unidirectional communication channel between one or many publishers and an arbitrary number of subscribers. Meanwhile, ROS service can implement the request–response paradigm with communication between clients and a single server. All theses messages to be exchanged can be standard ROS-defined or user-defined.

In this framework, information includes two kinds: the sensor information and the geometry information. For sensor information, currently we have:

- */motion_states* as *geometry_msgs/Pose*
- */joint_states* as *sensor_msgs/JointState*

All geometry information in ROS is managed by *tf* transformation library, including every robot parts, markers (marker array), sensors and hands.

### Model management

The motion planning framework called MoveIt [17] is used in our work to replace previous *robot_description* stack. With MoveIt Setup Assistant, we can build the configuration files for any given robot only if we have their 3D models files and physical parameters. In our case, we integrated one type of industrial robot, the Staubli TX90, with MoveIt. Meanwhile, for better kinematics performance, we use a Kinematics/IKFast [18] to automatically build kinematics plugin. Therefore, the model management will consist of two packages, the *moveit_robot_config* and *kinematics_plugins*.

### Manipulation control

To drive robot with motion sensing commands, the manipulation control module servers as an operator: pulling the published motion commanders and sending request to physical robot controller through a set of ROS actions and services.

**Fig. 9:**
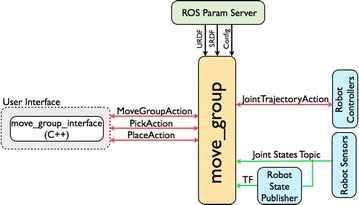
Manipulation control architecture. High-level architecture for the robot manipulation provided by MoveIt

When developing this module, we take advantage of the robot control architecture, called *move_group* in MoveIt. As shown in Fig. 9, *move_group* will look for configuration files in ROS param server to generate full robot model management, and communicate with the physical robot controller to get current state information (positions of the joints, etc.) and to send next state goals. For motion sensing operation, this control interface talks with physical robot controller using the *FollowJointTrajectoryAction* interface. Currently, in our framework, we implement *MoveGroupAction*, *Pick* and *Place* action client with roscpp through the following developed API for motion sensing control:

- *bool motion_setPose(geometry_msgs::Pose pose);*
- *geometry_msgs::Pose info_getPose();*
- *std::vector* $\langle double \rangle$ *info_get_joints();*

### Visualization

The ROS visualizer provides a tool for robotic manipulating scene and information display. Additionally, with Gazebo simulator [19], we can simulate robot in a real physical world with realistic sensor feedback and physically plausible interactions between objects. Part of these functions has been established in our previous work [20]. For friendly use, the ROS node */dashboard_gui* provides a simple dashboard-style dashboard user interface for operation and settings.

Therefore, our manipulation control module has realized the processes from subscribing position goals from */motion_command_publisher*, planning and generating executable trajectory, and finally publishing the path to the hardware interface.

## General hardware interface

The hardware interface layer is acting as translator between target hardware controller and ROS foundation. This layer is a combination of very low level communication protocols and software interfaces, enabling applications to access and operate physical robot.

In general, almost all modern industrial robot platforms provides interfaces for users to communicate with controllers that is the reason why ROS industrial was developed. Currently, industrial manipulators, like ABB, Kuka, Adept, Fanuc, Motoman, Staubli and Universal Robots, are supported by ROS-Industrial packages, and this length of list is still increasing with more developer creating standard interfaces to stimulate “hardware-agnostic” software development for new manipulators. In our research, we found that once a robot controller could provide access to position control (get and set positions of the joints or end effector), a hardware interface to operate this manipulator would be developed.

And in our study, we defined a standardized interface template with general service. And the standardized ROS service is defined in Table 2.

**Table 2:** Standardized ROS service for general hardware interface development

motion_commander/commander.srv	
uint8	command_index
std_msgs/Header	header
float32[]	position
float32[]	orientation
float32[]	joints
– – –	
uint8	result
std_msgs/Header	header
float32[]	position
float32[]	orientation
float32[]	joints

In practice, supporting communication protocols is relayed to the operation system running in physical robot controller. Generally, Ethernet and CAN bus are widely used. The hardware interface binds ROS and physical robot controller together. Trajectories from manipulator control module are streamed to the controller through the supported communication protocol. After that, controller buffers these points and interpolates between them to drive motors of the robot to the desired pose. And here we give the API template for general hardware interface development:

- *bool GetRobots(std::vector* $\langle int \rangle$ *robots);*
- *bool Login(const std::string url, const std::string userName, const std::string password);*
- *bool GetRobotJoints(std::vector* $\langle double \rangle$ *joints);*
- *bool GetRobotCartesianPosition(std::vector* $\langle double \rangle$ *position);*
- *bool SetJoints(const std::vector* $\langle double \rangle$ *joints);*
- *bool MoveL(std::vector* $\langle double \rangle$ *pos);*
- *bool MoveJ(std::vector* $\langle double \rangle$ *pos);*

The implementation of these classes is architecture dependent. In our framework, this layer is packaged and installed as robot drivers, which can be easily updated over the air using the built-in rosinstall mechanism, when adopting new manipulators.

After obtaining the connections between ROS and the targeted robot controller, we can build two nodes: *motion_controller_server* and *motion_controller_client* with defined messages types to establish the request–response paradigm for motion commands publishing.

## Implementation and experiments

### Experiment configuration

To preliminarily validate the framework, we conduct motion sensing manipulating experiments both in simulation and on physical robot. The motion sensing input devices are Kinect Xbox 360 and Leap Motion with SDK v2. The targeted robot is the 6 axis Staubli TX90 with the SOAP communication protocol supported. We can easily generate the *staubli_moveit_config* and *staubli_tx90_ikfast* packages. After that, we modify the launch files and start to check the scene in Rviz.

### Experiment result and analysis

**Fig. 10:**
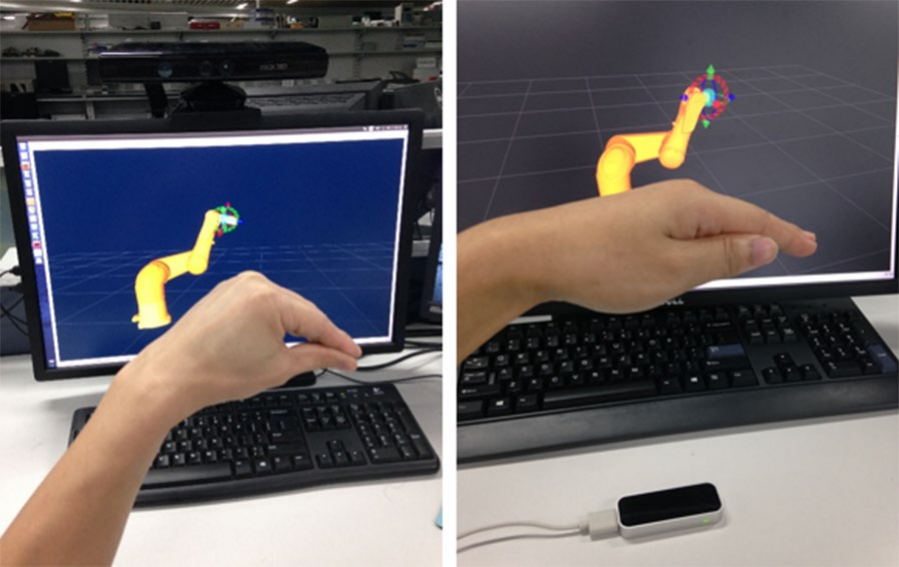
Motion sensing robotic manipulating using the proposed framework. With Microsoft Kinect (*left*) and with Leap Motion (*right*)

The result of the motion sensing-based robot manipulation experiment in simulation is shown in Fig. 10. Both Kinect and Leap Motion are tested under the same environment.

**Fig. 11:**
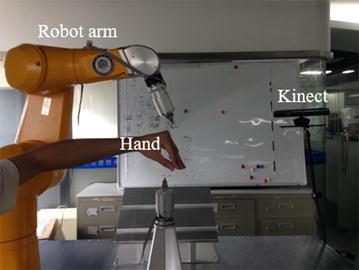
Motion sensing robotic manipulating on the physical robot with Kinect in which the robotic arm is moving from initial place to the first picking pose as the hand commands

**Fig. 12:**
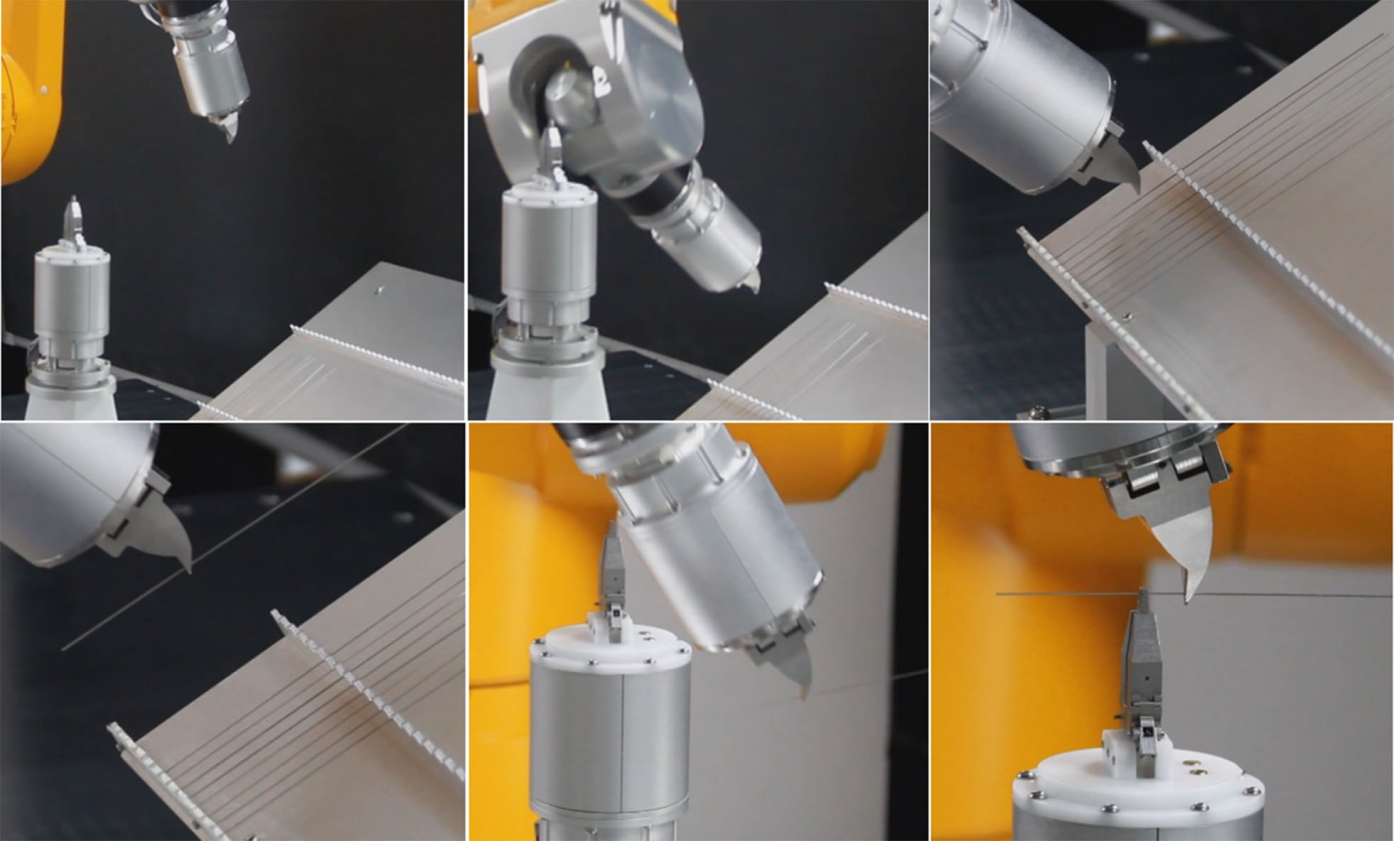
Snapshots of motion sensing robotic manipulating, moves from initial place to the first picking pose, picks the object and places at the desire position

And a very simple pick and place manipulation experiment was also conducted on the physical robot with Kinect, as setup in Fig. 11. We can easily teach the robot where the picking pose and placing pose are by the hand gestures captured from Kinect. The proposed framework will transform and command the robotic arm to desired positions. And Fig. 12 gives the snapshots of robot manipulation from initial pose to picking pose and the final placing pose.

The results have shown that the proposed framework is feasible for robotic manipulation with motion sensing control.

## Conclusion

This paper proposed a motion sensing-based robotic manipulation framework. The framework contains an integrated motion sensing devices driver for gesture commands recognition, a ROS-based framework core to map motion sensing intents to robot operation commands, and a general robot hardware interface for compatibility with varies robot manipulators. The validation in simulation and physical robot have shown that the proposed framework is feasible for robotic manipulation with motion sensing control. And hardware driver repository can be found here: https://github.com/pinkedge/ROS-I_hardware_drivers.git.

## Discussion and future work

The main contributions of our research are: (1) designing a modular motion sensing framework, which can bind brands of input devices and manipulators together, (2) proposing to use the ROS built-in rosinstall mechanism to update hardware interfaces over the air for general hardware compatibility. However, there are still some issues and improvements to be addressed in our future work: first, dynamic response and accuracy improvement. The performance variations between different motion sensors made it difficult for our algorithm to evaluate and take an unified control; second, smart and robust trajectory replication. In teach-pendant operation pattern, the system is a typical human-in-the-loop. With DTW spacing registration method, the requirement for precise trajectory imitation was not so high. Thus, our future works are: (1) quantitative analysis of dynamic behaviors and accuracy, (2) trajectory replication performance improvement, (3) application of dual-arm manipulation.
